# Proteomics-Identified Bvg-Activated Autotransporters Protect against *Bordetella pertussis* in a Mouse Model

**DOI:** 10.1371/journal.pone.0105011

**Published:** 2014-08-18

**Authors:** Daan de Gouw, Marien I. de. Jonge, Peter W. M. Hermans, Hans J. C. T. Wessels, Aldert Zomer, Alinda Berends, Catherine Pratt, Guy A. Berbers, Frits R. Mooi, Dimitri A. Diavatopoulos

**Affiliations:** 1 Laboratory of Pediatric Infectious Diseases, Department of Pediatrics, Radboud University Medical Centre, Nijmegen, The Netherlands; 2 Laboratory of Medical Immunology, Department of Laboratory Medicine, Radboud University Medical Centre, Nijmegen, The Netherlands; 3 Nijmegen Centre for Mitochondrial Disorders, Department of Laboratory Medicine, Radboud Proteomics Centre, Radboud University Medical Centre, Nijmegen, The Netherlands; 4 Netherlands Centre for Infectious Disease Control, National Institute for Public Health and the Environment (RIVM), Bilthoven, The Netherlands; 5 Public Health England, Centre for Emergency Preparedness and Response, Porton Down, Salisbury, United Kingdom; Instituto Butantan, Brazil

## Abstract

Pertussis is a highly infectious respiratory disease of humans caused by the bacterium *Bordetella pertussis*. Despite high vaccination coverage, pertussis has re-emerged globally. Causes for the re-emergence of pertussis include limited duration of protection conferred by acellular pertussis vaccines (aP) and pathogen adaptation. Pathogen adaptations involve antigenic divergence with vaccine strains, the emergence of strains which show enhanced *in vitro* expression of a number of virulence-associated genes and of strains that do not express pertactin, an important aP component. Clearly, the identification of more effective *B. pertussis* vaccine antigens is of utmost importance. To identify novel antigens, we used proteomics to identify *B. pertussis* proteins regulated by the master virulence regulatory system BvgAS *in vitro*. Five candidates proteins were selected and it was confirmed that they were also expressed in the lungs of naïve mice seven days after infection. The five proteins were expressed in recombinant form, adjuvanted with alum and used to immunize mice as stand-alone antigens. Subsequent respiratory challenge showed that immunization with the autotransporters Vag8 and SphB1 significantly reduced bacterial load in the lungs. Whilst these antigens induced strong opsonizing antibody responses, we found that none of the tested alum-adjuvanted vaccines - including a three-component aP - reduced bacterial load in the nasopharynx, suggesting that alternative immunological responses may be required for efficient bacterial clearance from the nasopharynx.

## Introduction

Pertussis (or whooping cough) is a highly contagious, acute respiratory disease of humans caused by the Gram-negative bacterium *Bordetella pertussis*. Although pertussis-related mortality has dropped significantly after the introduction of childhood vaccination, pertussis has resurged in many industrialized countries, with particularly large outbreaks occurring in 2010 and 2012 in Australia, the USA, the UK, the Netherlands, and several other countries [1]–[4]. This increase in pertussis incidence is seen mainly in (vaccinated) adolescents and adults, providing a reservoir for transmission of pertussis to unvaccinated or partially vaccinated newborns, who are at greatest risk of developing severe pertussis [5]–[7].

Although current pertussis vaccines are effective in limiting the development of severe clinical symptoms, they are much less effective in preventing colonization of the upper respiratory tract and consequently do not adequately reduce circulation in the population [8]. Moreover, vaccine-induced protection wanes rapidly leaving vaccinated individuals susceptible to develop disease after 5–7 years [9]. The apparent inability of current vaccines to significantly reduce the circulation of *B. pertussis* may have facilitated pathogen adaptation. Pathogen adaptation has resulted in antigenic divergence between vaccine strains and circulating strains and the emergence of strains, designate P3 strains, which show enhanced *in vitro* expression of a number of virulence-associated genes [10], [11]. Most recently, strains belonging to the P3 lineage have emerged which do not produce pertactin (Prn), a component of most aPs. Prn-deficient strains have reached frequencies of up to 55% in some countries [12]–[15]. Efforts to improve the immunogenicity of pertussis vaccines have thus far focused on skewing immunity towards more effective bacterial clearance, for instance through the use of novel adjuvants [16], [17]. However, the emergence of Prn-deficient strains also highlights a need to identify novel protective antigens, which may be included in improved aPs.

The expression of nearly all pertussis virulence factors, including the antigens present in aPs, is positively regulated by the two-component sensory transduction system BvgAS (reviewed in [18]). Bvg-activated proteins are generally associated with virulence and modulation or evasion of host immunity [19] and play an important, or even essential role, during infection. These proteins therefore represent potential vaccine targets. *In vitro*, low temperature and increasing concentration of nicotinic acid or sulfate are known to suppress the Bvg-system, resulting in the transition from virulent (Bvg^+^) through intermediate (Bvg^i^) to nonvirulent (Bvg^−^) bacteria [20]. In this study, we analyzed the Bvg^+^, Bvg^i^, and Bvg^−^ phase-dependent protein content of two clinical isolates derived from the P1 and P3 lineages which dominated globally *before* and *after* the 1990s, respectively [11], [21]. Conserved *in vivo* expressed candidates were then evaluated for their ability to confer protection against respiratory infection in mice.

## Results

### Proteomic analysis and vaccine antigen selection

**Figure 1:**
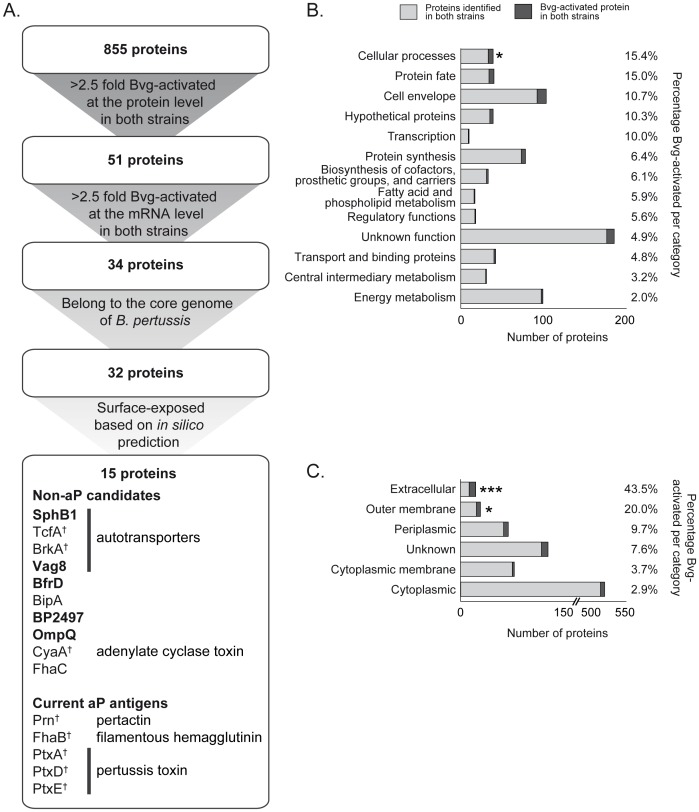
Vaccine antigen selection and functional clustering of Bvg-activated proteins. **A**) Putative protein antigens were selected based on ≥2.5-fold Bvg-activation at both protein (this work) and mRNA level [10], presence in the core genome of *B. pertussis* [25], and PSORTb v3.0 predicted surface accessibility (outer membrane or extracellular) [26]. This resulted in the identification of 15 putative vaccine candidates. † known *B. pertussis* protective antigens. Proteins in bold were selected for further examination. The proteins in bold The 855 and 51 proteins that were respectively identified and Bvg-activated at the protein level in both strains, were grouped by functional categories (**B**) and PSORTb-predicated subcellular localization (**C**). The relative frequency of Bvg-activated proteins compared to the total number of annotated proteins identified in both strains for each functional class are listed on the right-hand side. Asterisks indicate statistically significant enrichment of Bvg-activated proteins in a certain class as determined by Fisher's exact test. *p<0.05, ***p<0.0005.

The regulation of the Bvg-system is dependent on environmental signals, including free sulfate [20]. In this study, we compared the proteome of two currently circulating *B. pertussis* lineages under *in vitro* Bvg^+^ (low sulfate), Bvg^i^ (medium sulfate), and Bvg^−^ (high sulfate) conditions. *B. pertussis* strains B1917 and B1920 were used as representatives for the P3 and P1 lineages, respectively [21]. Bacterial cultures were grown in four replicates under different Bvg conditions, fractionated into cytosolic and membrane protein fractions and analyzed by mass spectrometry. This approach identified a total of 940 proteins in B1917 and 952 proteins in B1920, with 91% overlap between these strains (see Text S1 and Figure 1A), representing ∼28% of the predicted 3449 protein-coding ORFs in the *B. pertussis* genome [22]. The IDEAL-Q algorithm [23] was used to identify proteins that were ≥2.5 fold higher expressed under Bvg^+^ or Bvg^i^ conditions compared to the Bvg^−^ condition, which yielded 159 and 104 Bvg-activated proteins in B1917 and B1920, respectively (Table S1).

Since P3 and P1 strains both circulate in the population [24], novel antigens should target both lineages. We therefore first selected proteins that were Bvg-activated in both strains, resulting in 51 potential candidates (Figure 1A). Aggregation based on function and predicted subcellular localization showed that these 51 candidates were significantly enriched for proteins involved in cellular processes (n = 6, 15%), outer membrane proteins (n = 6, 20%) and extracellular proteins (n = 10, 44%; Figure 1B and C). Subsequent selection based on Bvg-dependent *in vitro* transcription levels [10], presence in the core genome of *B. pertussis* [25] and predicted surface-exposed localization [26] yielded 15 vaccine candidates (Figure 1A). Of these 15 proteins, eight represent known *B. pertussis* protective antigens (Figure 1A), thus validating our strategy to identify virulence factors, some of which have proven immunogenic properties. Of the remaining candidates, the autotransporters SphB1 and Vag8, the TonB-dependent receptor for iron transport BfrD, the zinc protease BP2497, and the outer membrane porin protein Q (OmpQ), were selected for further examination (Figure 1A). Although the outer membrane ligand binding protein BipA and FhaC are also promising vaccine candidates based on these criteria, their vaccine potential was not assessed due to limited resources.

The list of vaccine candidates was supplemented with proteins that were highly abundant under all Bvg conditions, as these may also represent attractive targets for the host immune response. Based on protein abundance estimation by emPAI [27] (Table S2), the two most abundant proteins, outer membrane proteins A and P (OmpA, OmpP), were also selected for further analysis.

### Recombinant protein production and vaccination

To analyze the protective potential of the selected vaccine candidates, recombinant His-tagged fusion proteins were expressed, purified, and refolded. BfrD and OmpQ could not be produced due to low expression and protein degradation. The five remaining proteins were adjuvanted with alum (5 µg for each antigen, 1 µg for SphB1 due to low protein yield) and administered to BALB/c mice (six mice per group) as stand-alone antigens by subcutaneous injection at day 0 and 14. In parallel, groups of mice were vaccinated with a 3-component aP or with PBS and/or alum. Three weeks after the final vaccination, mice were challenged by intranasal infection with *B. pertussis* strain B1917. B1917 was chosen as the challenge strain, as it belongs to the predominant P3 lineage, which has spread worldwide [11], [24]. Importantly, the amino acid sequences of the tested antigens are highly conserved in all sequenced *B. pertussis* strains [24].

**Figure 2:**
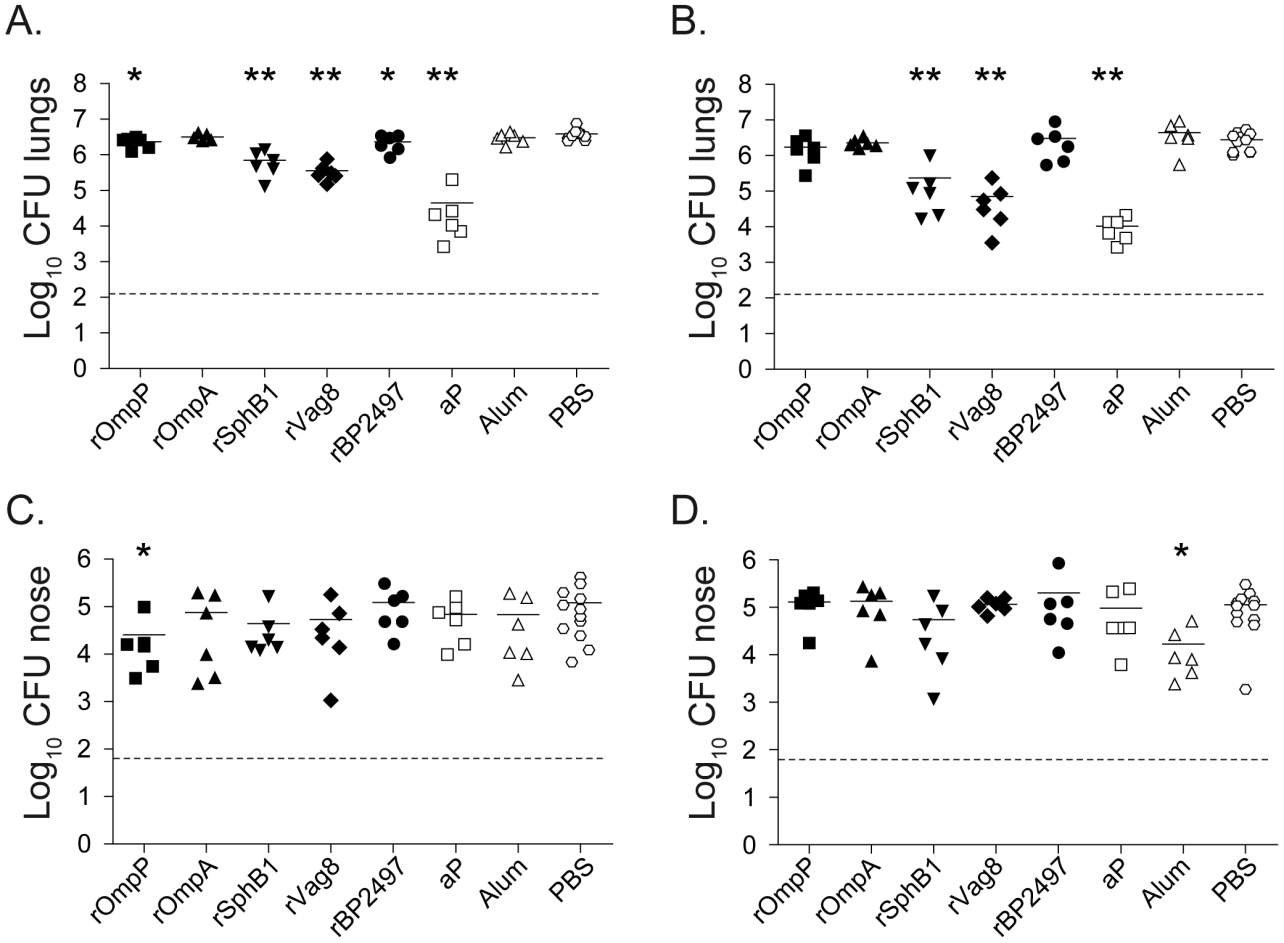
Effect of immunization with novel vaccine candidates on infection with *B. pertussis* in the lower and upper respiratory tract. Naïve adult female BALB/c mice were subcutaneously immunized as described in the text and infected intranasally with 2×10^7^ CFU of *B. pertussis* strain B1917. The bacterial load in the lungs and nose was quantified three (**A&C**) and seven (**B&D**) days after challenge. Each symbol represents one mouse. Horizontal lines represents the mean. Dashed lines indicate the lower limit of detection. *p<0.05, **p<0.005 relative to PBS mice; 2-tailed Mann-Whitney *U* test.

aP-vaccinated mice showed the strongest (>220-fold) reduction in bacterial load in the lungs at both 3 and 7 days after infection. Of the novel vaccine candidates, rOmpA did not confer any protection. Although vaccination with rOmpP and rBP2497 resulted in a small but significant reduction of bacterial numbers in the lungs at day 3 (1.7 and 1.8-fold, respectively), these differences were not observed at day 7, suggesting that these proteins have only limited vaccine potential (Figure 2A and B). In contrast, vaccination with either rSphB1 or rVag8 resulted in a nearly 10-fold reduction in bacterial load in the lungs at day 3 and a more pronounced 26 and 68-fold reduction at day 7, respectively (Figure 2A and B). Of note, although alum-vaccination induced a significant 7.3-fold reduction in bacterial load in the upper respiratory tract (URT) at day 7 compared to the PBS group, none of the vaccines, including aP, was able to reduce bacterial load in the URT (Figure 2C and D).

### Antibody responses to vaccine antigens

**Figure 3:**
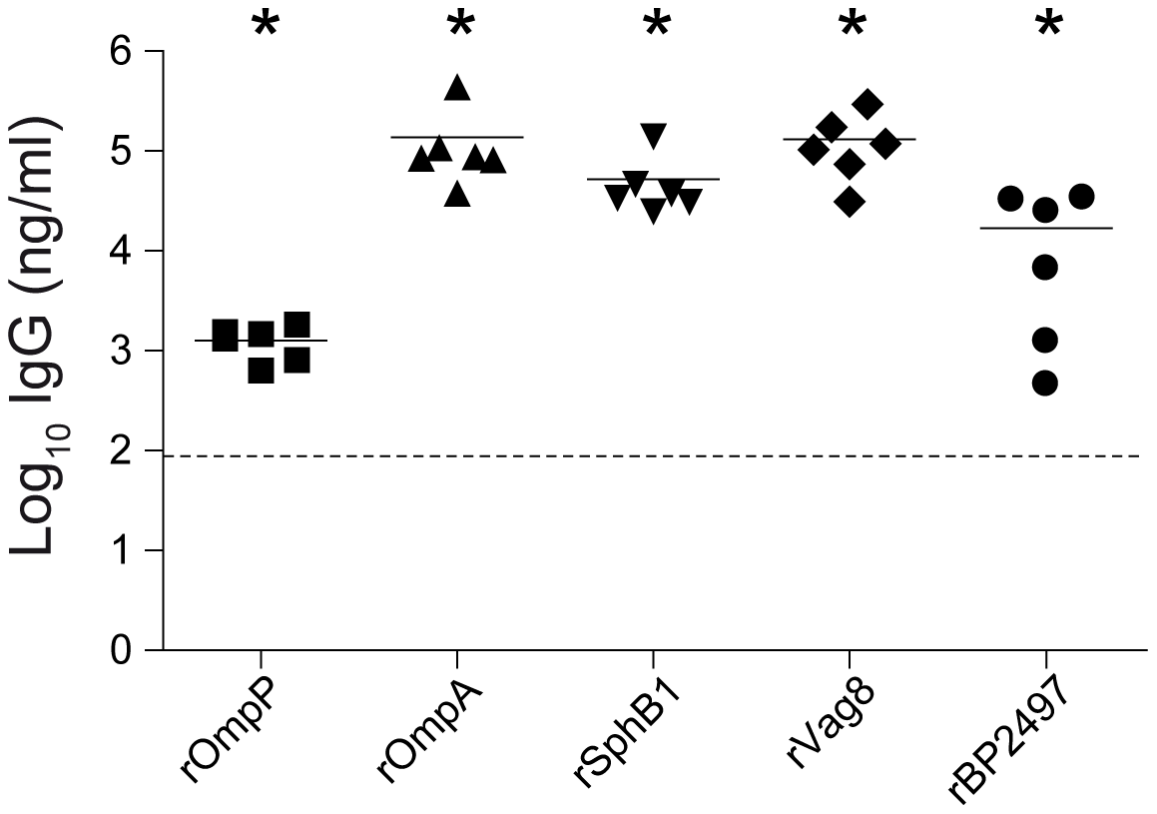
Detection of antigen-specific IgG in the serum of immunized mice. Post-immune serum (day 28) was used to determine the amount of total IgG specific for each recombinant antigens using ELISA as described in the text. Each symbol represents one mouse and the geometric mean is represented by a line. Dashed lines indicate the lower limit of detection (93 ng/ml). *p<0.05; 1-tailed Wilcoxon Signed Rank Test.

**Figure 4:**
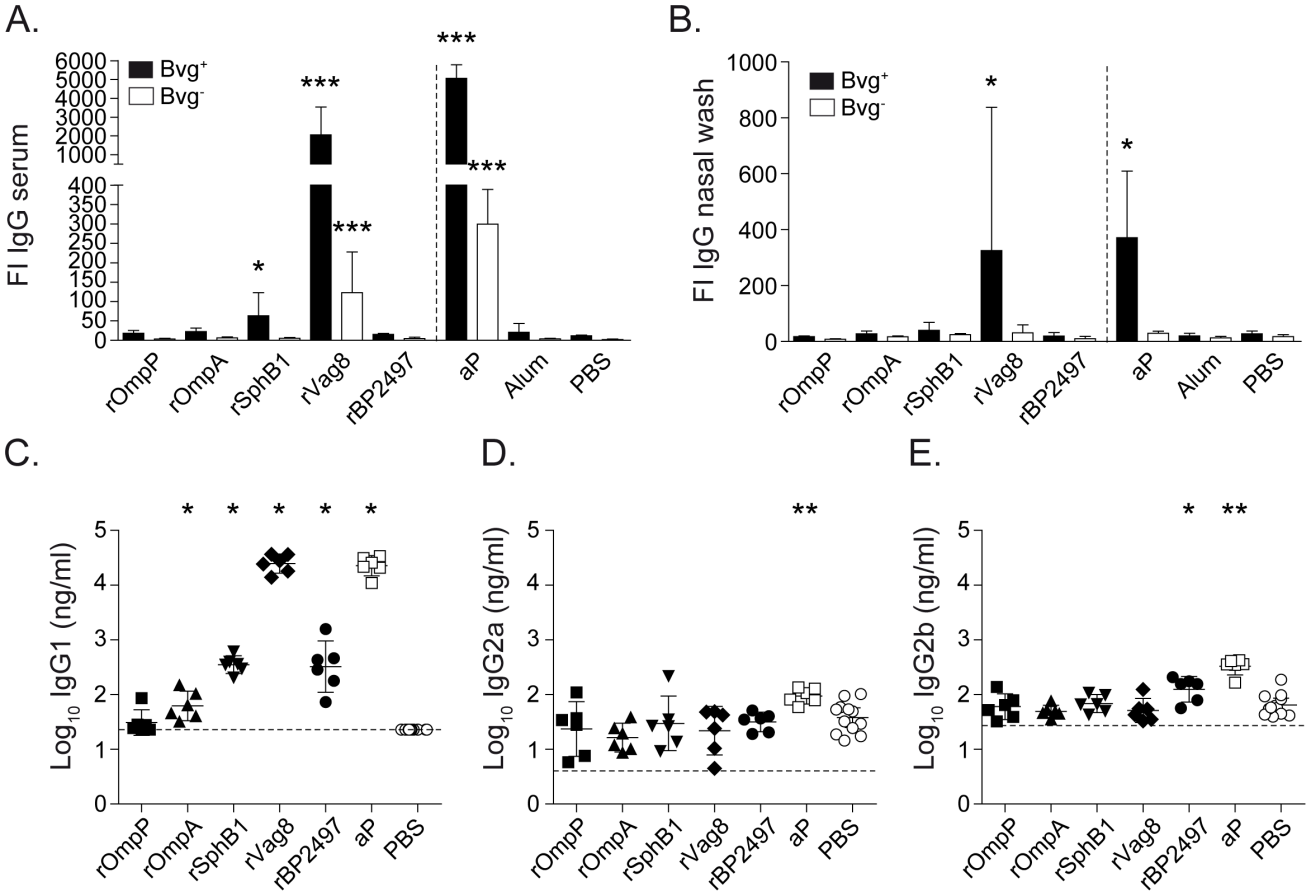
Antibody-mediated opsonization of *B. pertussis*. The binding of serum (**A**) and nasal lavage (**B**) IgG antibodies to Bvg^+^ or Bvg^−^ grown *B. pertussis* B1917 was determined using flow cytometry. Bars represent the geometric mean ±95% confidence interval (CI) of 6 individual mice. *p<0.05, ***p<0.0005 relative to PBS group; Kruskal-Wallis test followed by a Dunns *post-hoc* test (α = 5%). To determine the distribution of IgG subtypes, whole-cell ELISA was performed with the post-immunization serum samples to detect pertussis-specific IgG1 (**C**), IgG2a (**D**), and IgG2b (**E**). Each symbol represents one mouse and the geometric mean is represented by a line. Dashed lines indicate the lower limit of detection (23, 4, and 27 ng/ml for IgG1, IgG2a, and IgG2b, respectively). For IgG1, a 1-tailed Wilcoxon Signed Rank Test was performed because IgG1 levels of the PBS mice were below the detection limit. IgG2a and IgG2b levels were statistically compared to the PBS mice using a 2-tailed Mann-Whitney *U*-test. *p<0.05, **p<0.005.

To determine the potential role of antibodies in vaccine-mediated opsonization, ELISA was performed using sera from vaccinated mice. Figure 3 suggests that all vaccine candidates induced production of antigen-specific IgG. The absence of pre-existing antigen-specific antibodies was confirmed using pre-immune serum. To assess binding to native epitopes on the bacterial surface, flow cytometry was used to measure binding of antibodies in serum and nasal lavage to the challenge strain B1917 grown under Bvg^+^ and Bvg^−^ conditions. Serum antibodies from rVag8, rSphB1, and aP-vaccinated mice opsonized Bvg^+^ bacteria at significant levels. Although opsonization levels were reduced for Bvg^−^ bacteria, rVag8 and aP-vaccinated mice sera still bound significantly to Bvg^−^ bacteria (Figure 4A), which is most likely due to residual expression of these proteins under Bvg^−^ conditions (Table S2). For nasal lavage samples, binding to Bvg^+^ bacteria was only detected for rVag8 and aP-treated animals (Figure 4B).

Finally, whole-cell ELISA was performed for each antigen. Analysis of the subtype distribution of opsonizing IgG1, IgG2a, and IgG2b antibodies showed that IgG1 was the dominant subtype for each antigen (Figure 4C, D en E).

### *In vivo* expression of vaccine antigens

**Figure 5:**
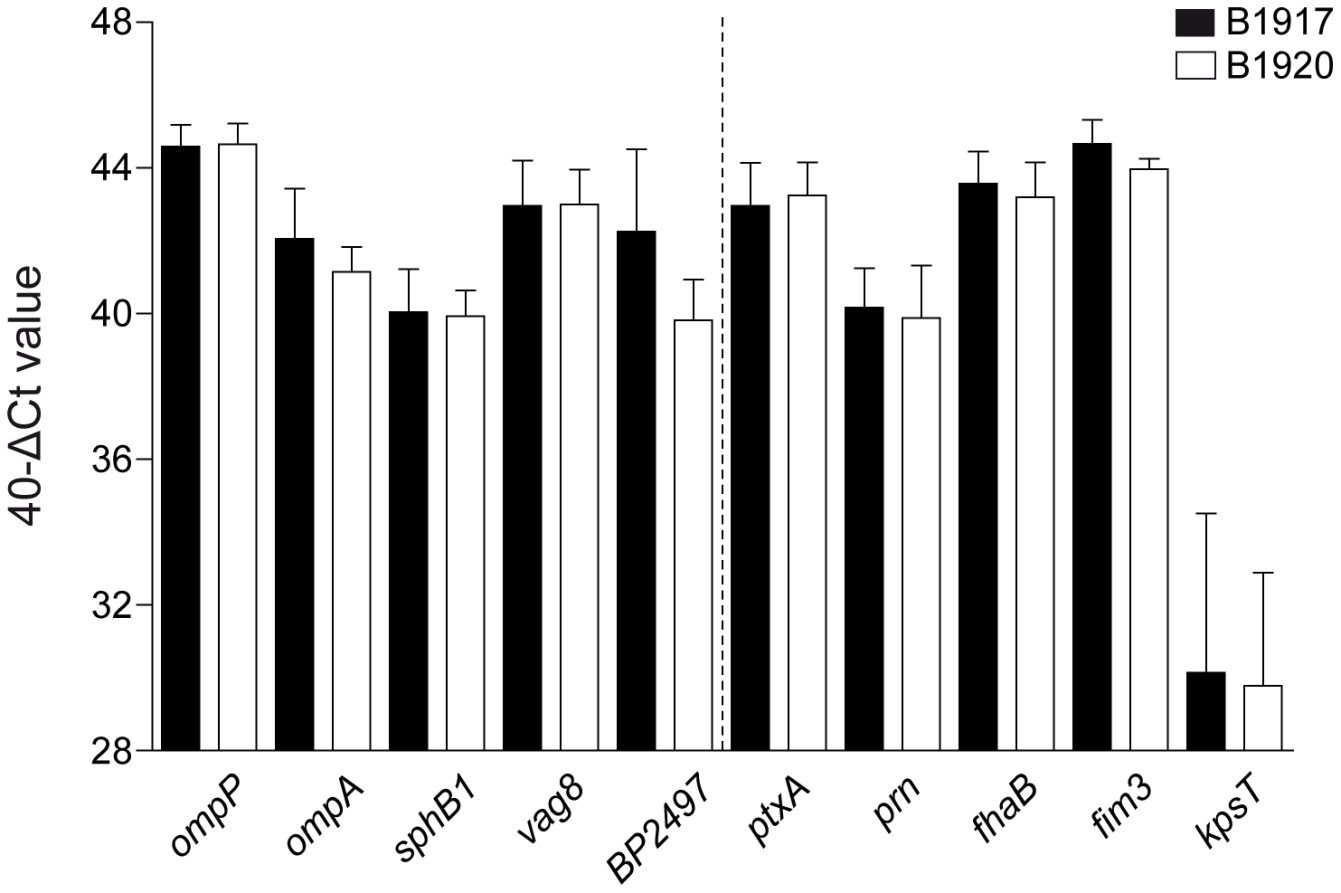
*In vivo* expression of vaccine-candidate genes. Naïve adult female BALB/c mice (n = 4) were infected intranasally with *B. pertussis* strain B1917 or B1920. After 7 days, bacteria were collected through broncho-alveolar lavage and used for *in vivo* transcriptional analysis using antigen-specific primers as described in the text. The transcription data is expressed as 40-ΔCt value, which is a measure of expression relative to the *recA* household gene (ΔCt  =  Ct *target* – Ct *recA*). The number 40 represents the number of PCR cycles. A 40-ΔCt value of 40 indicates that the gene is expressed at equal levels as *recA*, while higher values correspond to higher expression.

To determine whether the selected vaccine candidates were expressed during infection, naïve adult BALB/c mice were infected intranasally with B1917 or B1920. Seven days after infection, gene expression was analyzed on bacteria isolated from the lungs for the five vaccine candidate genes as well as for *ptxA*, *prn*, *fhaB*, and *fim3*, which encode all currently used aP antigens except for Fim2. The expression of these genes was compared to the *kpsT* gene, which encodes a protein involved in capsule biosynthesis that has previously been identified as a Bvg^−^ gene [28]. Transcriptional analysis showed that all vaccine candidates and aP antigens were highly expressed in the lungs of mice in relation to *kpsT* (Figure 5).

## Discussion

The increase in pertussis outbreaks and the many adaptations observed in *B. pertussis* populations, including the down-regulation of Prn, indicates that more potent pertussis vaccines are warranted. Here, using an integrated ‘omics’ approach, we identified OmpP, OmpA, SphB1, Vag8, and BP2497 as *in vivo* expressed vaccine candidates. Stand-alone immunization with the autotransporters SphB1 and Vag8 induced significant protection against lower respiratory tract (LRT) infection, at a level which was only 10- and 3-fold lower compared to the reference 3-component aP vaccine (containing Ptx, Prn, and FHA), respectively. Thus far, four *B. pertussis* autotransporters have shown to confer protection in the mouse model, Prn [29], TcfA [30], SphB1, and Vag8, suggesting that these proteins represent an attractive class of protective antigens.

Since aluminum adjuvants typically induce a strong T helper type 2 (Th2) response and high levels of antibodies [31], we primarily focused on the contribution of antibodies to protection. Although all five selected vaccine candidates induced significant levels of specific serum IgGs (predominantly IgG1), only rSphB1 and rVag8 conferred significant protection. A potential explanation for this result may be that only antibodies to rSphB1 and rVag8 opsonized *B. pertussis* (Figure 4). The inability of antibodies specific for rOmpP, rOmpA, and rBP2497 to successfully opsonize bacteria may be due to incorrect folding of the recombinant proteins, which is essential to induce bactericidal antibodies particularly to integral outer membrane proteins like OmpP and OmpA [32].

At present, the exact mechanisms of protection induced by SphB1 and Vag8 remain unknown. Antibodies to these proteins may facilitate phagocytosis and subsequent killing, or result in the deposition of complement factors on the bacterial surface. Alternatively, antibodies may neutralize the biological activity of these antigens. For instance, antibodies against Vag8 may enhance the susceptibility of *B. pertussis* to complement-mediated killing [33]. Interestingly, SphB1 induced similar protection levels compared as Vag8 (Figure 2), despite much lower opsonization levels (Figure 4). This could be a direct consequence of the lower absolute expression levels of *sphB1* during infection compared to *vag8* (Figure 5). Alternatively, the lower level of opsonizing antibodies may still be sufficient to effectively neutralize the biological activity of SphB1 on the bacterial surface. SphB1 is a serine protease which plays an essential role in the maturation of the adhesin and immune-modulating factor FHA [34]. Previous studies have shown that deletion of the *sphB1* gene dramatically attenuated the ability of *B. pertussis* to infect mice [35] and enhanced phagocytosis [36]. Taken together with our observation that *sphB1* is not expressed at high levels during infection, this gene represents a very attractive target because even low concentrations of neutralizing antibodies may be sufficient for protection.

Although we found that SphB1 and Vag8 were both expressed during infection in naïve mice, antibodies against these (recombinant) antigens were undetectable in convalescent pertussis patients (data not shown). Although it is possible that these proteins have poor intrinsic immunogenicity in humans during natural infection, another explanation may be that vaccinated individuals who are subsequently infected with *B. pertussis* preferentially respond to only a limited number of immunodominant vaccine antigens, also known as original antigenic sin [37].

Finally, an important observation was that none of the vaccine formulations, including aP, was able to reduce bacterial colonization of the URT. As colonization of the URT is probably essential for transmission, this may explain epidemiological studies which show that high circulation of *B. pertussis* occurs despite widespread aP vaccination [38]. Similarly, recent observations in the baboon model have shown that aP-vaccinated baboons are protected against disease but remain susceptible to colonization and are able to transmit the disease [8]. These data suggest that the immunological mechanisms which are required for effective clearance from the lungs (e.g. antibodies [39]) are distinct from those in the URT, an observation which has also been made for other respiratory bacterial pathogens, including *Streptococcus pneumonia* [40]–[42]. Cellular immunity in particular may represent an important component for effective clearance of the URT [43] and in order to prevent both disease *and* colonization, a combined antibody and cellular response may be required [8], [17], [44]–[47].

## Materials and Methods

### Ethics statement

Animal experiments were approved by the Radboudumc Committee for Animal Ethics and conducted in accordance with the relevant Dutch legislation. Bacterial strains were collected by regional Medical Microbiology Laboratories from patients suspected of whooping cough and sent to the National Institute for Public Health and the Environment (RIVM) in the context of routine surveillance (as required by law). The strains are sent to the RIVM for confirmation of clinical diagnosis, species determination and subtyping. Strictly anonymized patient information is included, which is limited to age, sex and postal code. For this type of surveillance ethical evaluation or patient consent are not required. The strains have been used in previous studies [48].

### Bacterial strains and growth conditions

Culture samples of *B. pertussis* strains grown under Bvg^+^, Bvg^i^, and Bvg^−^ conditions in chemically defined THIJS medium [49] from a previous study [10] were used for proteomic analysis (four replicates). For modulation of the BvgASR regulatory system, magnesium sulfate was added to cultures at a final concentration of 5 and 50 mM to induce Bvg^i^ and Bvg^−^ conditions respectively. In the absence of additional sulfate, the concentration of free sulfate was determined to be 0.02 mM, thereby inducing Bvg^+^ conditions. Correct modulation of the Bvg system by sulfate was confirmed in previous work [10]. Bacteria harvested at mid-log (OD_620_ of 0.5–0.6) were used for protein isolation.

For recombinant protein expression, *Escherichia coli* OverExpress C41(DE3) (Lucigen, USA) was grown in Luria Bertani medium (LB) containing appropriate antibiotics (ampicillin, kanamycin and/or chloramphenicol at a concentration of 50 µg/ml) at 37°C.

### Proteomics analysis

Bacterial pellets from 5 ml of mid-log culture were lysed by sonication. Cytosolic and membrane protein fractions were then isolated using the ReadyPrep Protein Extraction Kit Membrane I (Bio-Rad Laboratories, Hercules, CA, USA), according to the manufacturers protocol. The obtained soluble and insoluble fractions, containing the cytosolic and membrane (-associated) proteins respectively, were precipitated using the ReadyPrep 2-D clean-up Kit (Bio-Rad Laboratories). Protein pellets were then dissolved in 8 M urea in 10 mM Tris-HCl pH 8.0 and subjected to in-solution digestion and C18 reversed phase nano flow LC-MS/MS analysis as described in Text S1.

### Recombinant antigen production

The DNA sequences of the selected vaccine antigens lacking their N-terminal signal sequences and/or other targeting domains to allow cytosolic expression, were codon optimized for expression in *E. coli*, synthesized by GenScript (USA), and cloned into pET28-TEVsite vector (modified from pET28a vector, Novagen, Denmark) to generate N-terminally His_6_-tagged proteins (Table S3). *E. coli* OverExpress C41(DE3) (Lucigen, USA) was cultured to an OD_600_ = 0.6–0.7 at 37°C, after which recombinant protein expression was induced through the addition of 1 mM Isopropyl β-D-1-thiogalactopyranoside (IPTG) for 3 hours. For cell lysis, bacteria were sonicated in Bacterial Protein Extraction Reagent (B-PER, Thermo Scientific). Recombinant proteins in inclusion bodies were dissolved in 20 mM sodium phosphate, 500 mM NaCl, 4 M urea and 20 mM imidazole and purified on a AKTA FPLC system using affinity chromatography on a HisTrap FF crude 1 ml column prepacked with Ni Sepharose 6 Fast Flow (GE Healthcare, Sweden) as described [50]. Protein concentrations were determined using the 2-D Quant Kit (GE Healthcare, USA). Recombinant proteins were refolded by rapid 50-fold dilution in refolding buffer (for rBP2315 and rBP2497 according to [51] and for rBP0840 and rBP0943 according to [52]) Refolding of rBP0216 was performed in 10 mM Benzamidine, 1 mM EDTA, 100 mM NaCL, 1 M urea, and 50 mM diethanolamine (pH9.5).

### Vaccination

Individual groups of six naïve female, 6–8 week old BALB/c mice (Charles River) were immunized subcutaneously on day 0 and 14 with 5 µg of recombinant protein (1 µg for rBP0216 due to low protein yield) mixed 1∶1 in 1.3% alum adjuvant (Alhydrogel; Sigma). As a control, mice were immunized with PBS and Alhydrogel, or with PBS alone. Since the PBS group was the most used group for statistical comparisons, 12 mice were included in this group to enhance the statistical power. In order to compare efficacy to current aPs, one group of mice was immunized with 1/50^th^ of the human dose (equals 1.16 µg of *B. pertussis* protein) of the commercial hexavalent 3-component acellular pertussis vaccine *Infanrix* (GSK, Belgium). On day 35, mice were challenged by intranasal (i.n.) infection with 2×10^7^ colony forming units (CFU) of *B. pertussis* strain B1917 in 40 µL. Bacterial load in the nasopharynx and lungs was determined on day 38 and 42 (3 and 7 days after challenge) as described previously [50]. Including the 7 day time-point allowed comparison to aP vaccination, which in mice typically leads to clearance of the pathogen from the lungs within 7 days [17]. Furthermore, the 3 day time point allowed us to determine whether the selected antigens are able to induce early protection against infection. Serum samples were collected on days 0, 28, 38, and 42.

### *In vivo* transcriptional analysis

Groups of 4 female, naïve 6–8 week old BALB/c mice (Charles River) were infected i.n. with *B. pertussis* strain B1917 or B1920 as described above. After 7 days (at the peak of infection), bacteria were collected from the lungs through a bronchoalveolar lavage (BAL) with PBS and stabilized with RNA Protect Bacteria Reagent (Qiagen). Total RNA was extracted using the RNeasy Mini kit (Qiagen) and contaminating genomic DNA was removed by DNase treatment (DNAfree, Ambion). Bacterial RNA was enriched and amplified using the MICROBEnrich (Ambion) and SensationPlus FFPE Amplification (Affymetrix) kits, respectively. Enriched RNA was reverse-transcribed using the SuperScript One-Cycle cDNA Kit (Invitrogen) and used for quantitative real-time PCR analysis (primer sequences available on request). To determine relative expression levels, ΔCt values were calculated by subtracting the Ct value of the *recA* (BP2546) household gene from the Ct value of the target gene [53]. The transcription data are expressed as 40-ΔCt value, with 40 representing the number of PCR cycles as detection limit. A 40-ΔCt value of 40 indicates that the gene is expressed at equal levels as *recA*, while higher values correspond to increased expression.

### Antibody analysis

#### Protein ELISA

IgG titers against the recombinant antigens in mouse and human sera were determined by sandwich enzyme-linked immunosorbent assay (ELISA) analysis, essentially as described previously [50].

#### Whole-cell ELISA

The binding of IgG subtypes to whole bacteria was measured using a whole-cell ELISA method adapted from Abdillahi and Poolman [54]. Briefly, ELISA plates were coated with Bvg^+^ mid-log culture of the challenge strain B1917, washed with PBS containing 0.05% Tween 20 (PBST), blocked with 1% BSA/PBS, and incubated with mouse serum. Bound IgG1, IgG2a, and IgG2b was detected using anti-mouse secondary antibodies (BD Pharmingin and Southern Biotech) and appropriate substrates. The optical density was measured on an ELISA plate reader (Tecan Infinite F50) and antibody subtype concentrations were determined by comparison to standard curves with known concentrations of each IgG subtype.

#### Opsonization

1% BSA/PBS was used for all dilutions. 10^6^ CFU of Bvg^+^ and Bvg^−^ B1917 (challenge strain) were incubated with serum or nasal lavage (NL) samples from vaccinated mice for 30 min at 4°C. Bacteria were fixed in 2% paraformaldehyde and surface-bound IgG was detected using anti-mouse IgG-Fc-FITC-conjugated antibodies (Sigma-Aldrich) on a BD LSRII flow cytometer (BD Biosciences). The amount of surface-bound antibodies was expressed in arbitrary units as a fluorescence index (FI), calculated by multiplying the geometric mean fluorescence intensity by the percentage of FITC-positive bacteria [55]. Data were analyzed using FlowJo version 7.6.5.

### Statistical analyses

A 2-tailed Mann-Whitney *U* test was used for comparison of bacterial load in NL and lung homogenate between PBS-vaccinated mice and recombinant antigen-vaccinated mice. A Kruskal-Wallis test followed by a Dunns *post-hoc* test (α = 5%) was used for comparison of antibody-mediated opsonization by serum and NL samples between PBS-vaccinated mice and recombinant antigen-vaccinated mice. A 1-tailed Wilcoxon Signed Rank Test was used to determine whether ELISA measured IgG levels were significantly above the detection limit. All statistical analyses were performed using the GraphPad Prism software program, version 5.0, where *p<0.05* was considered significant.

## Supporting Information

Table S1 Proteomics data of statistically significant Bvg-regulated proteins identified in the cytosolic and membrane fraction of *B. pertussis* strains B1917 (P3) and B1920 (P1).
(XLSX)

Table S2 Protein abundance of all proteins identified in the cytosolic and membrane fraction of *B. pertussis* strains B1917 (P3) and B1920 (P1).
(XLSX)

Table S3 Constructing His-fusions of the selected candidate antigens.
(XLSX)

Text S1 **Supplemental Methods.**
(PDF)
