# Correction: Proteomics-Identified Bvg-Activated Autotransporters Protect against *Bordetella pertussis* in a Mouse Model

**DOI:** 10.1371/journal.pone.0113322

**Published:** 2014-11-06

**Authors:** The PLOS ONE Staff

The names of the first and second authors are incorrect in the citation. The correct citation is: de Gouw D, de Jonge MI, Hermans PWM, Wessels HJCT, Zomer A, et al. (2014) Proteomics-Identified Bvg-Activated Autotransporters Protect against *Bordetella pertussis* in a Mouse Model. PLoS ONE 9(8): e105011. doi:10.1371/journal.pone.0105011

## References

[1] Gouw Dd, Jonge MId, Hermans PWM, Wessels HJCT, Zomer A, et al (2014) Proteomics-Identified Bvg-Activated Autotransporters Protect against *Bordetella pertussis* in a Mouse Model. PLoS ONE 9(8): e105011 doi:10.1371/journal.pone.0105011 25133400 10.1371/journal.pone.0105011 PMC4136822

