# Supplementary material for: Proteomics-Identified Bvg-Activated Autotransporters Protect against Bordetella pertussis in a Mouse Model
Source: PLoS One. 2014 Aug 18;9(8):e105011. doi: 10.1371/journal.pone.0105011 (PMC4136822; doi:10.1371/journal.pone.0105011)
Supplement: Text S1 — Supplemental Methods. (PDF) [file pone.0105011.s004.pdf]

TEXT S1- SUPPLEMENTAL METHODS

In-solution digestion

Cytosolic and membrane protein extracts (15 µg) present in 8M urea in 10 mM Tris-HCl pH 8.0 were subjected to disulfide reduction. For this, 1 µL 10 mM dithiothreitol (DTT) was added and incubated for 30 min at RT. Then, proteins were alkylated by adding 1 µL of 50 mM of iodoacetamide (IAA) in 50 mM Ammonium bicarbonate (ABC) and incubating for 20 min at RT. After digestion with Endoproteinase LysC (1:50 w/w) (Wako Chemicals, USA) for 3 hours at RT, the solution was diluted to 2M urea with ABC and incubated overnight in the presence of sequencing grade modified trypsin (1:50 w/w) (Promega, Madison, WI) at 37 ^{o}C for complete digestion. Subsequently, the peptide mixture was concentrated and desalted using stop and go elution (STAGE) tips as described by Rappsilber and coworkers [1]. Briefly, the acidified peptides were bound on activated C18 reversed phase material fixed in a standard pipettor tip, washed with 0.5% acetic acid and eluted with 80% acetonitrile 0.5% acetic acid. Finally, the acetonitrile buffer was removed using a SpeedVac (Savant DNA 110 SpeedVac; Global Medical Instruments) after which the peptides were dissolved in 0.5% acetic acid for nLC-MS/MS analysis.

C18 Reversed phase LC-MS/MS analysis

The C18 reversed phase nanoflow LC-MS/MS analyses were performed using a Proxeon nanoflow liquid chromatograph (Easy-nLC, Thermo Fisher, Bremen, Germany) coupled on-line to a 7T linear quadrupole ion trap Fourier transform ion cyclotron resonance mass spectrometer (LTQ-FT Ultra, Thermo Fisher Scientific, Bremen, Germany) as described [2]. Briefly, chromatographic separations were performed using a 15-cm fused silica emitter (PicoTip emitter; tip, 8 ± 1 µm; internal diameter, 100 µm; FS360-100-8-N-5-C15; New Objective) packed in-house with reverse phase ReproSil-Pur C18AQ 3 µm resin (Dr. Maisch GmbH). Peptides were eluted from the column using a linear gradient of 8-16% acetonitrile in

75 min and 16-32% acetonitrile in 55 minutes at a flow rate of 300 nL/min. The LTQ-FT ultra mass spectrometer was programmed to acquire a survey MS scan in the ICR cell (350-1600 m/z, R=100.000 FWHM @ 400 m/z, 1E6 ions) with parallel MS/MS spectra acquisition of the top 4 most abundant ions in the linear ion trap (30% normalized collision energy, 30ms activation time, activation Q=0.250, 3000 ions). Dynamic exclusion was enabled to prevent re-analyses of peptides during the analysis (exclusion duration: 180 seconds, early expiration enabled: 10 scans with S/N=2). Only ions with charge states z=2+ and 3+ were considered for MS/MS spectra acquisition

Database searching and result validation

Database searches and validation of results was performed as described [2]. Briefly, the raw data files acquired by the nLC-MS/MS instrument were converted to mascot generic files using DTA supercharge [3]. Peptides and proteins were identified using Mascot software (Mascot 2.2; Matrix Science) to search a local copy of an in-house created pan-genomic *B. pertussis* protein database (based on described sequence data [4]) supplemented with known contaminant protein sequences (e.g. Trypsin, LysC and human skin proteins). The following parameters were used: 15 ppm precursor mass tolerance and a 0.5 Da fragment ion mass tolerance. Furthermore, one missed trypsin cleavage was tolerated and carbamidomethylation of cysteines was set as a fixed modification. Variable modifications included oxidation of methionine residues and N-terminal protein acetylation. The Mascot search results were subjected to heuristic iterative protein false discovery rate validation method of Weatherly to achieve a 1% false discovery rate or better [5]. A protein was considered identified when it was detected in at least 3 out of 4 biological replicates of at least one Bvg-condition. Exponentially modified PAI (emPAI) scores were calculated [6] to determine protein abundance in the different samples.

Label-Free Quantitative analysis

The IDEAL-Q (ID-based Elution time Alignment by Linear regression Quantification) software program was used for the label-free quantitative analysis of the nLC-MS/MS data [7]. The cytosolic and membrane protein fractions of both strains were processed independently. Semi-quantitative information was extracted from the LC-MS data and the Mascot search results by extracted ion currents. Details of the IDEAL-Q method are described by Tsou and coworkers [7]. Briefly, IDEAL-Q attempts to extract ion current information for every identified peptide (identified in any analysis) even in the absence of Mascot identification data for a peptide in some of the analyses. The following peptide ion information is used by IDEAL-Q to pinpoint ions across files where no identification information is available: mass to charge ratio (m/z), charge state (z), normalized retention time, and isotopic pattern. The combination of all of the above mentioned peptide ion characteristics allow IDEAL-Q to identify ions when no Mascot data is available for a specific analysis. Settings used by IDEAL-Q include 30 ppm mass tolerance, nondegenerate unique peptides only, and Dixon's outlier test to eliminate peptide ratio outliers for each proteins at 95% significance level. Protein ratios were calculated as the weighted average of all respective peptide ratios.

Processing semi-quantitative proteomics data

Protein abundance ratios were generated by IDEAL-Q for each biological replicate relative to Bvg^- sample one and normalized using the median ratio of all proteins quantified. The normalized ratios were Log2 transformed and used for a One-Way ANOVA with the maximum number of permutations (=34650) to identify proteins that were significantly different (p-value $\leq$0.05) between the three groups. Proteins that were found to be significantly different based on less than three out of four biological replicates in one of the groups that were significant were excluded. The p-values of all the remaining proteins (both significant and non-significant) were corrected for multiple testing using the FDR method of

Storey and Tibshirani to obtain q-values [8]. Proteins with a q-value ≤ 0.05 and a fold change of ≥3 or ≤-3 were considered as Bvg-regulated. Due to the high complexity of our (fractionated) protein samples and the limited capacity of the mass spectrometer to detect and quantify every peptide from every protein in all samples, quantitative information about the complete proteome was not available in our proteomic datasets. For some proteins quantitative information was lacking or highly variable, making it impossible to determine whether these proteins were Bvg-regulated. To partially overcome this limitation, we compared both the Bvg^+ and Bvg^i groups to the Bvg^- group and considered proteins that were at least 3-fold up or downregulated in either the cytosolic fraction, the membrane fraction, or both fractions, as Bvg-regulated. Bvg-regulated proteins were aggregated based on function (main role according to the TIGR *B. pertussis* genome and *B. bronchiseptica* database) and subcellular localization (predicted using PSORTb v3.0 [9]) and significant enrichment in a certain class was determined by Fisher's exact test.

Validation proteomic datasets

Proteomics allowed us to identify a total of 940 proteins in P3 and 952 proteins in P1, representing 28% of the total predicted ORFs in the P3 and P1 genomes. 855 proteins (91%) were identified in both strains. Of the 940 proteins identified in the P3 strain, 253 (27%) proteins were found only in the cytosolic fraction, 164 (17%) only in the membrane fraction, and 523 proteins (56%) were identified in both fractions. In the P1 strain, 613 of the 952 proteins (64%) were identified in both fractions whereas 181 (19%) and 158 (17%) proteins were found uniquely in the cytosolic and membrane protein fraction respectively.

Correct fractionation of the cytosolic and membrane proteins was confirmed using the protein abundance emPAI values of proteins with a strongly predicted cytosolic and outer membrane localization (determined using PSORTb v3.0). This revealed clear enrichment (high emPAI scores) for cytosolic predicted proteins in the cytosol fractions and membrane-

predicted proteins in the membrane fractions of all conditions in both strains (data not shown). Furthermore, western blot analysis revealed that the outer membrane protein BP0840 was exclusively present in the membrane protein fraction of all samples (data not shown). This data indicates correct protein fractionation of all samples.

Supplemental references

1. Rappsilber J, Ishihama Y, Mann M (2003) Stop and go extraction tips for matrix-assisted laser desorption/ionization, nanoelectrospray, and LC/MS sample pretreatment in proteomics. Anal Chem 75: 663-670.
2. Wessels HJ, Gloerich J, van der Biezen E, Jetten MS, Kartal B (2011) Liquid chromatography-mass spectrometry-based proteomics of Nitrosomonas. Methods Enzymol 486: 465-482.
3. Pedrioli PG (2010) Trans-proteomic pipeline: a pipeline for proteomic analysis. Methods Mol Biol 604: 213-238.
4. Bart MJ, van Gent M, van der Heide HG, Boekhorst J, Hermans P, et al. (2010) Comparative genomics of prevaccination and modern *Bordetella pertussis* strains. BMC Genomics 11: 627.
5. Weatherly DB, Atwood JA, 3rd, Minning TA, Cavola C, Tarleton RL, et al. (2005) A Heuristic method for assigning a false-discovery rate for protein identifications from Mascot database search results. Mol Cell Proteomics 4: 762-772.
6. Ishihama Y, Oda Y, Tabata T, Sato T, Nagasu T, et al. (2005) Exponentially modified protein abundance index (emPAI) for estimation of absolute protein amount in proteomics by the number of sequenced peptides per protein. Mol Cell Proteomics 4: 1265-1272.

7. Tsou CC, Tsai CF, Tsui YH, Sudhir PR, Wang YT, et al. (2010) IDEAL-Q, an automated tool for label-free quantitation analysis using an efficient peptide alignment approach and spectral data validation. Mol Cell Proteomics 9: 131-144.

8. Storey JD, Tibshirani R (2003) Statistical significance for genomewide studies. Proc Natl Acad Sci U S A 100: 9440-9445.

9. Yu NY, Wagner JR, Laird MR, Melli G, Rey S, et al. (2010) PSORTb 3.0: improved protein subcellular localization prediction with refined localization subcategories and predictive capabilities for all prokaryotes. Bioinformatics 26: 1608-1615.
